# Gene–environment interactions and vitamin D effects on cardiovascular risk

**DOI:** 10.1186/s12916-019-1402-x

**Published:** 2019-08-30

**Authors:** Guido Iaccarino, Bruno Trimarco

**Affiliations:** Department of Advanced Biomedical Sciences, Federico II University, Naples, Italy

**Keywords:** Vitamin D, Genetics, Environment, Cardiovascular risk, Supplementation, Parathormone

## Background

Mendelian randomization is the random assortment of genes from parents to offspring that occurs during gamete formation. It represents a type of natural randomization in large populations, where the combination of the variants of one or more genes determine the intensity of a phenotype [1]. Plasma 25-hydroxyvitamin D (pVitD) concentrations are affected by polymorphisms of *DHCR7* and *CYP2R1* genes, such that clustering a population according to their haplotypes allows the stratification of lifelong exposure to low or high pVitD levels. Such a natural experiment was employed by Afzal et al. [2] to show that, in a large European cohort, lifelong exposure to reduced pVitD levels is associated with an increased risk of all-cause mortality.

In the human quest for immortality, this evidence would imply that vitamin D supplementation is a simple way to increase life expectancy – a type of longevity elixir. However, results from clinical trials that have challenged this issue remain inconclusive, in particular those assessing the effect of vitamin D supplementation on cardiovascular-related deaths as well as the risk of cardiovascular events [3]. In this scenario, Huang et al. [4] further hamper the expectations of vitamin D supplementation enthusiasts. The authors confirm that genetically elevated pVitD levels, when examined in a comprehensive analysis of European and Han Chinese populations, do not provide protection against cardiovascular events. However, the manuscript presents additional interesting points of discussion.

### Comparison of European and Chinese cohorts

The study by Huang et al. [4] combined the results of two extensive studies in Europe (Copenhagen City Heart Study (CCHS) and the Copenhagen General Population Study (CGPS)) and one in China (China Kadoorie Biobank (CKB)). However, the European and the Chinese cohorts obviously differ regarding their genetic background as well as age, sex, body mass index, concurrent morbidities, consequential therapies, and overall and mean pVitD levels. Indeed, the latter was shown to be 50% higher in the Chinese than in the European cohorts (84 nM/L vs. ~ 50 nM/L). Although the authors did not provide a definitive explanation for this difference, it is easily interpreted by a possible role of non-genetic determinants of pVitD levels such as sun exposure, food intake, microbiome, and age. Nevertheless, regardless of the cause for the difference in pVitD levels between the European and Chinese cohorts, Huang et al.’s study [4] showed a difference in the incidence of cardiovascular events in the CCHS and CGPS studies versus that in the CKB cohort. The cardiovascular risk (CVR) in Europeans was shown to be affected by the combined genetics of *DHCR7* and *CYP2R1*, with those that had genetically determined lower pVitD levels experiencing an increased number of cardiovascular events. Furthermore, the changes in pVitD levels caused by Mendelian clustering were more significant in the European sample compared to the Chinese cohort, confirming that the interaction between genetics and environment plays a role in the final phenotype. Dietary habits or sun exposure trends of the Chinese population are likely to compensate for the genetic effects on pVitD levels, while in northern Europeans, these environmental effects are less meaningful. Moreover, many other studies in the general populations of Southern Europe, including ours in Southern Italy [5], suggest that, in Europe, the range of pVitD levels in the general population is broader than that shown in the CKB cohort. Therefore, in Europe as whole, there is a broader range of pVitD levels than in the CKB study. Given the more substantial difference, it is possible to deduce that, among Europeans, CVR phenotypes are more sensitive to extremely low pVitD levels.

### Vitamin D and its physiological role

Vitamin D plays a role in many homeostatic mechanisms in physiology, in particular in calcium metabolism. Low levels of vitamin D interfere with calcium reabsorption and are associated with increased parathormone levels (PTH). PTH and CVR are directly correlated with such a correlation being consistently observed in studies involving both primary and secondary hyperparathyroidism [6, 7]. The underlying pathophysiology is in turn attributed to different mechanisms, from increased cardiac damage [8] to endothelial dysfunction [9]. At every age, pVitD and plasma PTH levels are inversely correlated, so that reduced levels of vitamin D can cause hyperparathyroidism and thus lead to an increased risk of cardiovascular events [5]. It is therefore possible that the CCHS and CGPS populations, being older and with a broader range of pVitD levels, presented with higher levels of PTH, leading to an increased risk of cardiovascular events. On the contrary, the Chinese cohort showed higher and more homogeneous pVitD levels, and such a stabilizing effect might have then prevented the identification of any effect on CVR.

## Conclusions

In the future, a better definition of the normal ranges of pVitD that also considers the interaction with other mechanisms of CVR, such as PTH, might help to depict a more detailed scenario and more precise impact on CVR. This definition is needed to help us identify those populations for which vitamin D supplementation would indeed be a beneficial therapeutic intervention.
